# The Adverse Effects of Auditory Stress on Mouse Uterus Receptivity and Behaviour

**DOI:** 10.1038/s41598-017-04943-8

**Published:** 2017-07-05

**Authors:** Zahra Jafari, Jamshid Faraji, Behroo Mirza Agha, Gerlinde A. S. Metz, Bryan E. Kolb, Majid H. Mohajerani

**Affiliations:** 1 Department of Neuroscience, Canadian Centre for Behavioural Neuroscience, University of Lethbridge, Lethbridge, AB T1K 3M4 Canada; 2 School of Rehabilitation Sciences, Iran University of Medical Science (IUMS), Tehran, Iran; 3 Golestan University of Medical Sciences, Faculty of Nursing & Midwifery, Gorgan, Iran

## Abstract

Stress during gestation has harmful effects on pregnancy outcome and can lead to spontaneous abortion. Few studies, however, have addressed the impact of gestational stress, particularly auditory stress, on behavioural performance and pregnancy outcome in mice. This study aimed to examine the effect of two types of gestational stress on uterus receptivity and behavioural performance. Pregnant C57BL/6 mice were randomly assigned to either auditory or physical stress conditions or a control condition from gestational days 12–16. The auditory stress regimen used loud 3000 Hz tone, while the physical stressor consisted of restraint and exposure to an elevated platform. Three behavioural tests were performed in the dams after weaning. Uterine receptivity was investigated by counting the number of sites of implantation and fetal resorption. Also, the offspring survival rates during the early postnatal period were calculated. Auditory stress caused an increase in anxiety-like behaviour, reduced time spent exploring new object/environment, and reduced balance when compared to the physical stress and control groups. Auditory stress also caused higher rates of resorbed embryos and reduction of litter size. Our results suggest that the adverse effect of noise stress is stronger than physical stress for both uterus receptivity and behavioural performance of the dams.

## Introduction

The nervous system has high resilience to the influence of diverse components of the maternal environment during fetal development. The noxious effects of maternal environmental factors such as alcohol, drugs, smoking, and stress cause significant stress on fetal development in humans and experimental animals^1–4^. Specifically, environmental aversive stimuli such as noise exposure play a critical role in shaping brain and behaviour^5^. In addition to the harmful effect of gestational stress on offspring’s brain structure and function, the adverse effect of stress during pregnancy on the female’s physiology and behaviour can persist even beyond parturition^6–8^. Furthermore, stress during gestation can constantly affect endocrine state, body weight^9, 10^, and maternal performance in pregnant and lactating laboratory rodents^6, 11, 12^.

Clinical studies show higher incidences of autoimmune diseases, allergies, infections, and cancer following stressful situations^13–15^. The adverse effect of stress on reproductive function has been reported in many studies^16–20^. For instance, the effect of stress on implantation and fetal growth during pregnancy can lead to spontaneous abortion^16, 20, 21^. Stress also alters the immune system in mammals, and altered immune function is likely a principal cause of spontaneous abortions^14, 16, 22^. Moreover, exposing pregnant mice to a short period of ultrasonic-acoustic stress early in gestation can significantly increase the rate of spontaneous abortion^14, 16, 20^. Such an increase in abortion rate could be the result of inhibition of protective suppression and promotion of tumor necrosis factor-alpha (TNF-alpha) release in the uterus decidua^16, 20^.

Infanticide is observed in approximately 35 to 50% of adult male mice and rats and 10% or less in adult females^23–25^. Although numerous studies have demonstrated detrimental effects from crowded housing conditions, daily handling, forced swimming, loud noise, heat and bright light, and physical restraint on pregnancy outcome in rodents^20, 26, 27^, the effects of gestational stress on the loss of pups due to eating or killing them by mothers in the early postnatal period is unknown. Pregnant mice in previous studies were typically euthanized at a specific gestational age, to study the effect of gestational auditory stress on uterus receptivity by counting the number of implanted and resorbed embryos^16, 18, 19^. Therefore, the impact of auditory stress during pregnancy on loss of pups in the first postnatal days remains unclear.

Auditory stress is often defined as an unwanted and unpleasant auditory stimulus that leads to physiological, behavioural, and biochemical changes in humans and nonhuman animals. High levels of acoustic stimulation (90–105 dB) severely impair the hypothalamic–pituitary–adrenal (HPA) axis activation in both dams and offspring^28^. In the present study, we examined the effects of two types of stress, specifically auditory stress (AS) and physical stress (PS), on pregnancy outcome and postpartum behaviours of mice. We acquired several measures of pregnancy outcomes for analysis including weight gain during pregnancy, pregnancy duration, the number of implantation sites, the number of reabsorption signs on the uterus, and the number of live and lost pups. To address the influence of gestational stress on behaviour, we employed three behavioural tests to examine animals cognitive function, motor control and balance performance: novel object recognition (NOR) test^29, 30^, elevated plus maze (EPM)^31–35^, and balance beam test (BBT)^36^. Although there are many behavioural tests designed to score animals behaviour in mice, we used these behavioural tests for two main reasons. First, they require no training, external motivation, or reward enabling us to monitor signs of implantation sites and resorbed embryos within a short time after weaning. Second, their ability to show the effects of stress on rodents’ behaviour has been previously well documented^36–39^. The NOR test, which assesses recognition memory, is widely used for investigating a wide range of cognitive, memory, and neuropsychological functions^30, 40, 41^. The EPM is a popular measure of laboratory rodent anxiety levels, where the difference between time spent in the open and closed arms provides an index of anxiety-like behavior in mice^31, 32, 42^. Similarly, the BBT is a sensitive test for early detection of balance deficits in rodents^43^. In view of the high rate of abortion in mice exposed to AS in the past studies^16, 18–20^, we hypothesised that AS would have a more detrimental effect on both pregnancy outcome and behavioural performance compared to PS.

## Results

The ages of the female mice were similar across the groups (F_2,27_ = 0.697, p = 0.507). Since no significant differences were observed between the two control groups in any of the measures used in this study (p > 0.05), the results of the control groups were pooled together.

### Behavioural tests

#### Novel object recognition (NOR) test

**Figure 1:**
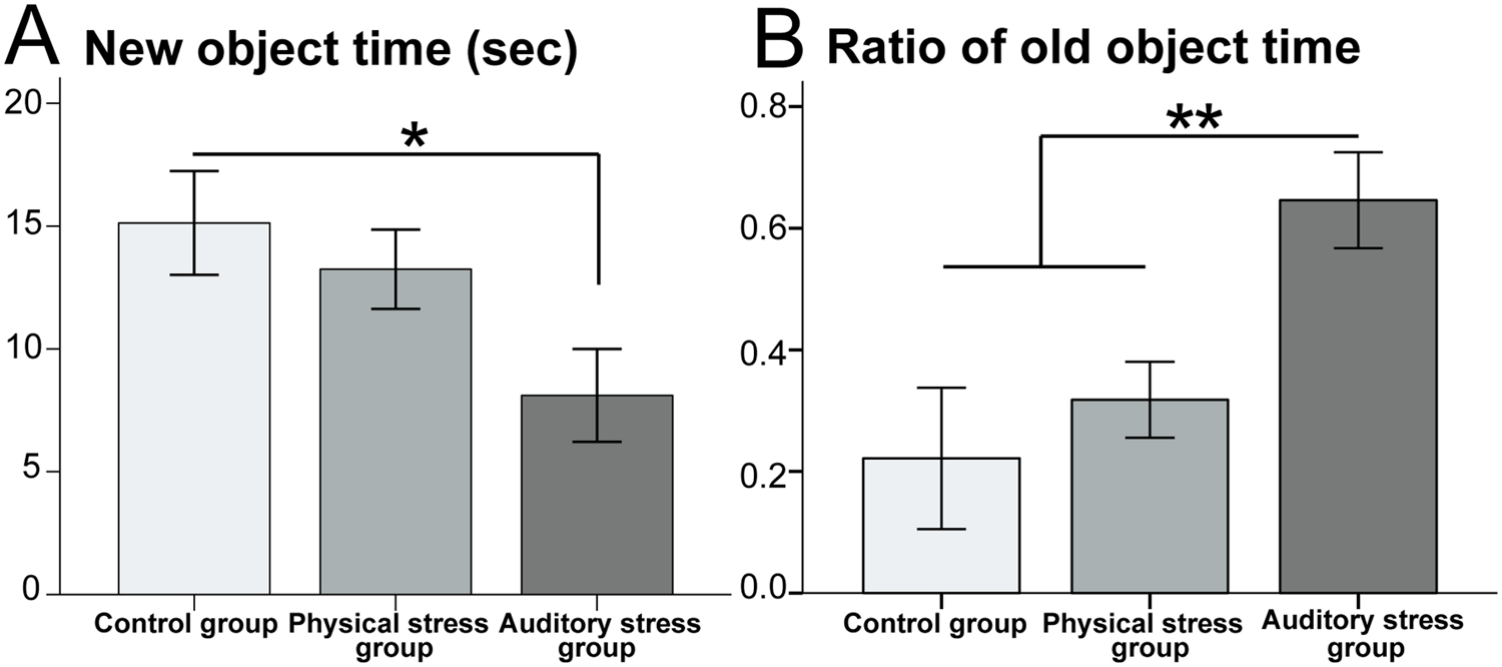
The novel object recognition (NOR) test: (**A**) the auditory stress group significantly spent shorter time with the new object compared with the control group. (**B**) The ratio of time spent with old compared with new object was significantly higher in the auditory stress group than the two other groups. N = 10 in all groups. Results are reported as mean ± S.E.M. Asterisks indicate *p < 0.05 or **p < 0.01.

Mice exposed to the AS spent significantly less time (sec) with the new object compared to those mice in the control group (Fig. 1A). Mice in both stress groups (AS and PS) spent more time with the old object compared to those animals in the control group but the difference was not statistically significant (F_2,27_ = 2.526, p = 0.101, η^2^ = 0.114). The ratio of time spent with old compared to the new object was significantly higher in the AS group than the two other groups (Fig. 1B). This experiment thus suggests that the gestational exposure to auditory stress reduces the dams’ ability to distinguish a new object from one that has been encountered previously (Table 1).

**Table 1:** Comparison among the three groups in different measures of the three behavioural tests.

Behavioural tests NOR test	*Between groups’ p-values			**Significant main effects		
	Control and PS	Control and AS	PS and AS	F	p	η^2^
New object time (sec)	0.480	**0**.**015**	0.065	3.583	**0**.**042**	0.198
Ratio of old object time (%)	0.432	**0**.**002**	**0**.**010**	6.663	**0**.**005**	0.358
**EPM test**						
Open arm time (sec)	0.203	**0**.**002**	**0**.**020**	6.767	**0**.**005**	0.370
Number of entries to open arm	0.139	<**0**.**001**	**0**.**002**	11.955	<**0**.**001**	0.489
**BBT**						
Latency (sec)	0.371	**0**.**004**	**0**.**019**	5.704	**0**.**010**	0.341
Number of foot slips	0.483	**0**.**014**	**0**.**041**	4.073	**0**.**031**	0.262
Number of turns	1.0	**0**.**009**	**0**.**005**	6.015	**0**.**008**	0.343

AS: auditory stress, BBT: balance beam test; EPM: elevated plus maze; NOR: novel object recognition, PS: physical stress, η^2^ = estimate of effect size. *The “between groups’ p-values” show p-values for the between group comparisons, and **“significant main effects” indicate the statistical results of a significant main effect for every measure.

#### Elevated plus maze (EPM) test

**Figure 2:**
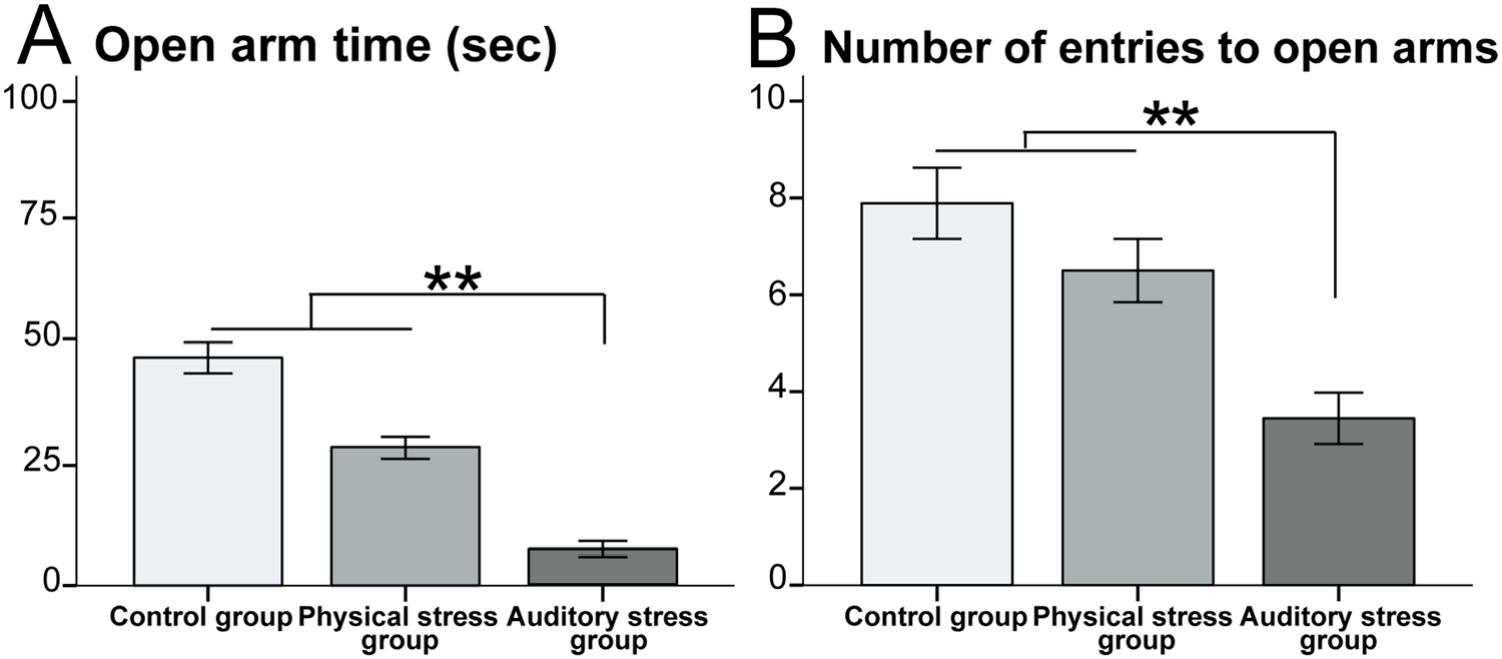
The elevated plus maze (EPM) test: The auditory stress group indicated (**A**) shorter time in open arms (sec), and (**B**) lower number of entries to open arm compared with the two other groups. N = 10 in all groups. Results are reported as mean ± S.E.M. Asterisks indicate *p < 0.05.

Table 1 shows that the AS group spent significantly less time (sec) in the open arms and had a lower number of entries to open arms, when compared to the other two groups. Figure 2 compares means of variables in all groups. This experiment thus demonstrates the anxioselectivity of the gestational exposure to auditory stress on dams on plus maze anxiety.

#### Balance beam test (BBT)

**Figure 3:**
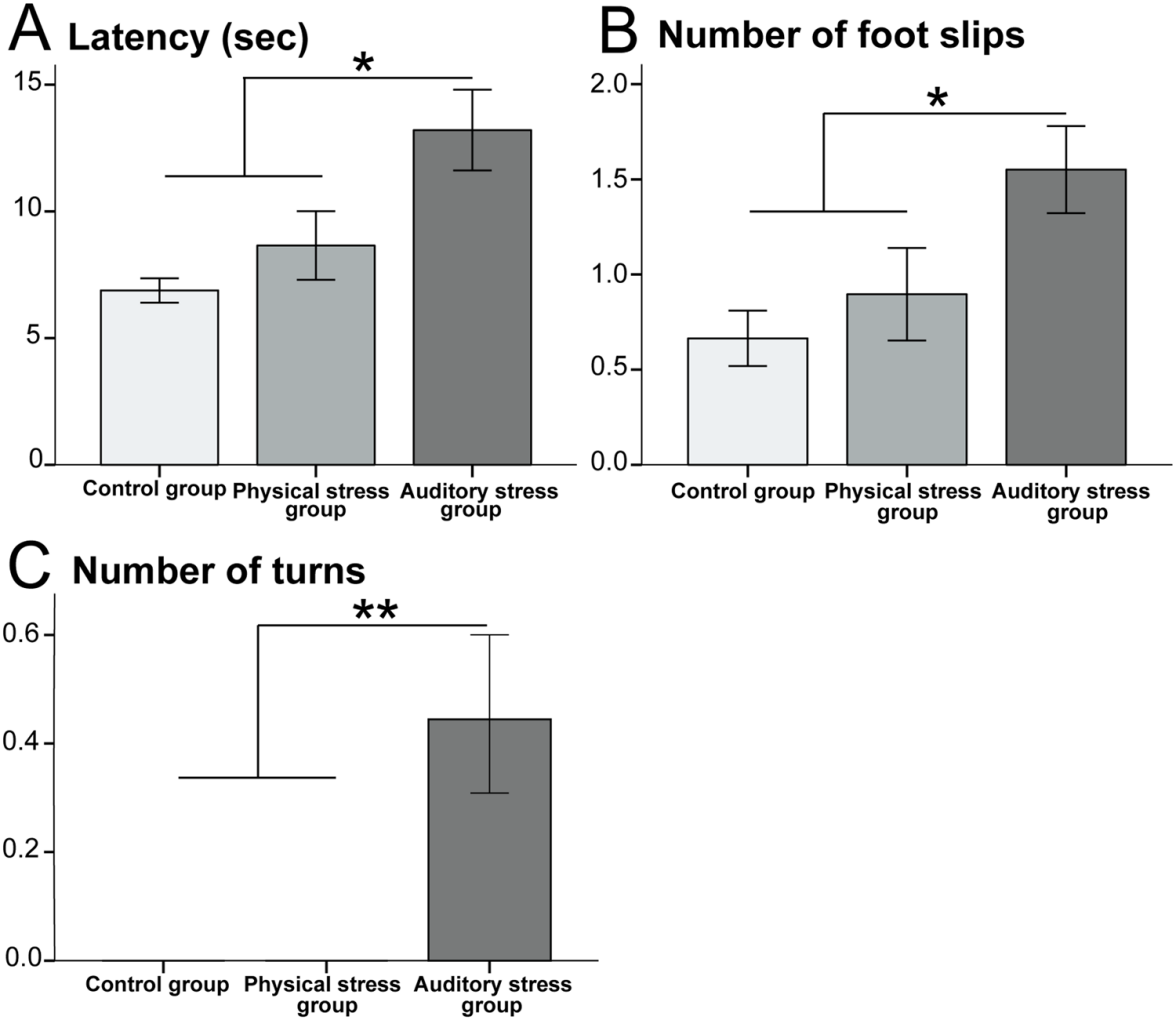
The balance beam test (BBT): The auditory stress group revealed (**A**) longer latency to travel across the beam (sec), (**B**) higher number of foot slips, and (**C**) higher number of turns compared with the two other groups. N = 10 in all groups. Results are reported as mean ± S.E.M. Asterisks indicate *p < 0.05 or **p < 0.01.

The AS dams were slower in crossing the beam (latency to cross, sec), had higher number of foot slips, and higher number of turns compared to the other groups (Table 1). Figure 3 illustrates summary results for measured variables among the three groups.

#### Mouse uterus receptivity and pregnancy outcomes

**Figure 4:**
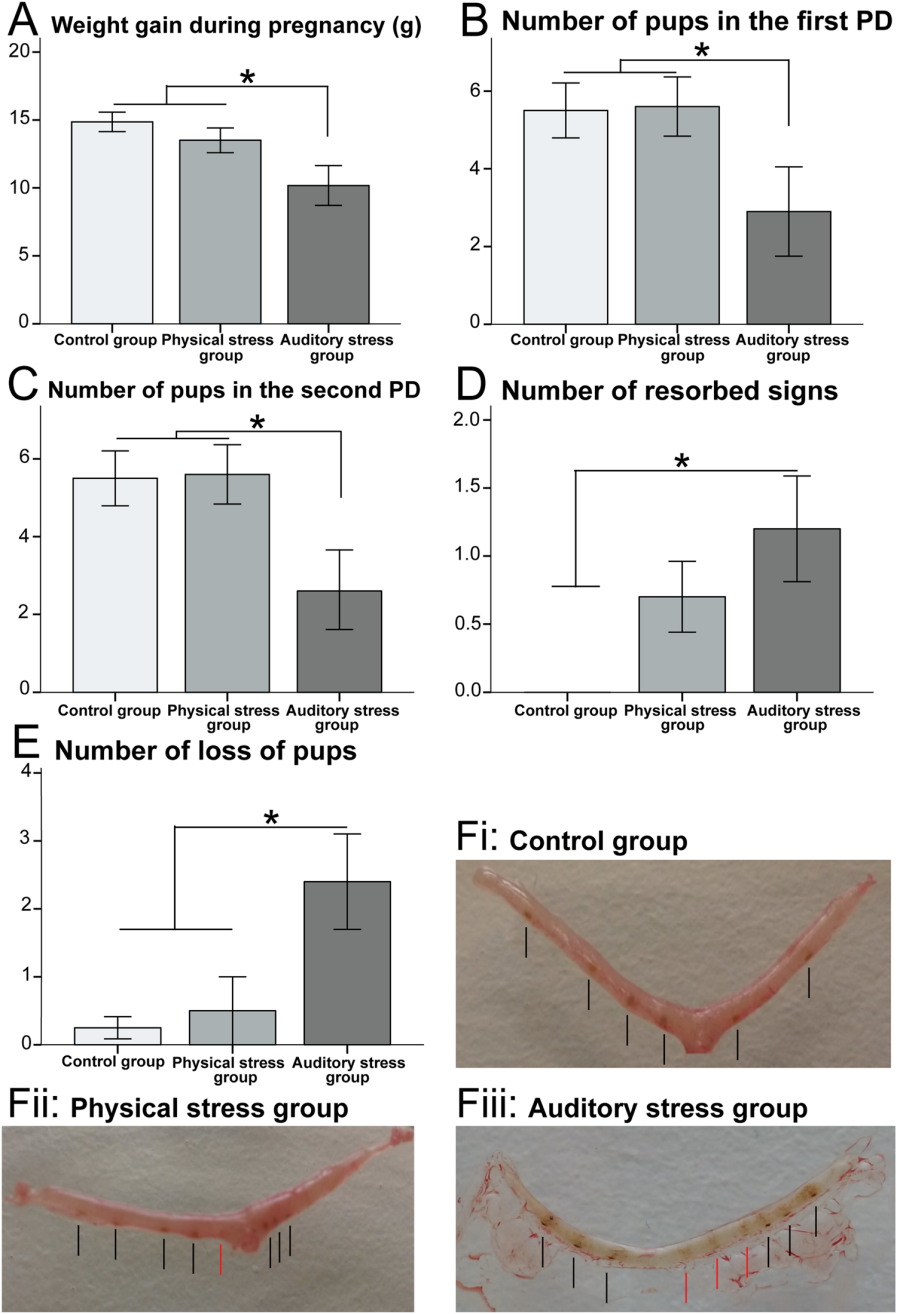
Pregnancy outcome: The auditory stress group obtained (**A**) lower weight gain during pregnancy (g), (**B**) decreased number of pups on the first postnatal day (PD), (**C**) fewer pups on the second PD, (**D**) higher number of resorbed signs, and (**E**) higher number of lost pups. N = 10 in all groups. Results are reported as mean ± S.E.M. Asterisks indicate *p < 0.05. *Uterus receptivity*: (**F**) A sample of marked uterus from each study group (fi = control, fii = physical stress, fiii = auditory stress). Black lines show the implantation sites and red lines show the resorbed signs.

Pregnancy duration was the same across all three groups (control group mean: 21.80 ± 0.91 day, PS group mean: 21.30 ± 1.33 day, and AS group mean: 21.5 ± 0.97 day; F_2,27_ = 0.531, p = 0.594, η^2^ = 0.038). Table 2 provides a summary of the statistical analyses of uterus receptivity and pregnancy outcome among the three groups. The dams in the AS group had significantly lower weight gain during pregnancy than either the PS or control dams (Fig. 4A). The number of live pups on the first PD (Fig. 4B), as well as the number of live pups on the second PD (Fig. 4C) was significantly lower in the AS group than the other groups. The difference between the number of pups on the first and the second PDs was not significant (F_2,27_ = 2.103, p = 0.159, η^2^ = 0.087). Moreover, the number of pups did not change after the second day of birth. Although the number of observed uterus implantation sites were similar in the three groups (control group mean: 5.78 ± 2.11; PS group mean: 6.1 ± 1.44, and AS group mean: 5.0 ± 2.22, F_2,27_ = 0.715, p = 0.498, η^2^ = 0.053), the number of resorbed signs, and the number of lost pups was significantly higher in the AS group compared to the other two groups (Fig. 4D–F). The difference between the number of surviving pups and the number of implantation sites was significant in the AS group (control group: F_1,9_ = 1.011, p = 0.343, η^2^ = 0.100; PS group: F_1,9_ = 2.250, p = 0.168, η^2^ = 0.200; AS group: F_1,9_ = 11.676, p = 0.008, η^2^ = 0.565). Figure 4Fi-iii, shows representative examples comparing the implantation sites and the reabsorption signs of uteri from each of the groups.

**Table 2:** Comparison among the three groups in terms of pregnancy outcome and uterus receptivity.

Uterus receptivity & pregnancy outcomes	*Between groups’ p-values			**Significant main effects		
	Control and PS	Control and AS	PS and AS	F	p	η^2^
Weight gain (g)	0.417	**0**.**008**	**0**.**041**	5.509	**0**.**011**	0.312
Number of pups on the first PD	0.941	**0**.**048**	**0**.**034**	3.446	**0**.**045**	0.212
Number of pups on the second PD	0.941	**0**.**038**	**0**.**022**	3.733	**0**.**046**	0.243
Number of resorptions	0.109	**0**.**009**	0.219	4.650	**0**.**030**	0.245
Number of loss of pups	0.753	**0**.**011**	**0**.**017**	4.802	**0**.**017**	0.278

AS: auditory stress, PD: postnatal day, PS: physical stress, η^2^ = estimate of effect size. *The “between groups’ p-values” show p-values for the between group comparisons, and **“significant main effects” indicate the statistical results of a significant main effect for every measure.

Overall, the PS group showed a trend toward the same pattern of results as the AS group (Figs 1–4), but the differences with the control group were not significant (p’s > 0.05).

## Discussion

Pregnant mice were assessed on three behavioural tests to investigate the effects of gestational acoustic and physical stress on exploratory, cognitive, and balance abilities on the dams. To evaluate recognition memory^30^ in our control and stressed groups, we used the novel object recognition (NOR) test. In the NOR test, the dams in the AS group spent significantly less time with the new object than the old one when compared to the control and PS groups. This reduced tendency for exploring the new object in the AS group suggests that the gestational exposure to auditory stress may cause a cognitive memory impairment. The EPM measures anxiety and was designed based on the competing natural tendencies of rodents to explore novel places and their innate behaviour to avoid unprotected, bright, and elevated places. The AS dams spent significantly less time in the open arms and showed a lower number of entries into open arms^44^. The reduced number of entries into open arms and time spent in the open arms in the AS mice indicates higher anxiety levels than the PS and control groups^31, 32, 35, 44, 45^.

Noise exposure can markedly produce behavioural and biochemical changes in both animals and humans. In general, subjecting animals to stress causes the dysregulation of HPA axis and can lead to various types of mood disorders, such as depression and anxiety^15, 31, 46, 47^. Specifically, high levels of environmental noise correlates with the psychological symptoms and the occurrence of psychiatric disorders^48^. Furthermore, similar to other types of stress, the AS can increase levels of stress hormones such as corticosterone and norepinephrine^49–51^. In recent decades, there is growing evidence showing detrimental effects of the long-term outcomes of the gestational stressful experiences on the psychological functioning of offspring^8^. Moreover, various epidemiological studies in humans demonstrate that stress during pregnancy increases the risk for anxiety- and depression-related disorders in children^15, 52–57^. Hence, the results from the NOR and EPM in the present study are consistent with previous literature, and demonstrate the long lasting effects of gestational stress on the mental wellbeing of mothers.

Balance behaviour is a complex ability that requires vestibular, skeletal muscle, and cerebellar-circuits for motor control. Abnormal physiological functions of these key systems are influenced by aging, structural damage, and genetic factors and can lead to balance-related dysfunction (e.g., deficits in posture, foot placement, and targeting)^58^. However, there is little information regarding how balance ability is affected by exposing mice to gestational stressors. In the present study, we investigated the crossing time, the number of foot slips and number of turns to assess balance and motor coordination. Mice in the AS group showed negative impacts in all measures compared to the PS and control groups. These results are consistent with a previous finding^36^ indicating that chronic exposure to low frequency noise at moderate levels (100 Hz with 70 dB SPL) impairs balance coordination in mice. The authors proposed that the effect was largely due to vestibular abnormalities. In view of the few studies regarding the effect of noise stress during gestation on the motor and balance coordination in the dams and offspring, we suggest investigations into the influence of noise characteristics (i.e., intensity, frequency spectrum, and duration) on balance performance as well as neuroendocrine system in mice.

The negative effect of gestational stress on reproductive function is a topic of interest that has been previously well-documented^14, 16, 18, 19^. Loud noise and physical restraint may represent stressful conditions that can influence pregnancy outcomes in rodents^16, 20^. Stress, for instance, has detrimental effects on fertility, mating behaviour, ovulation, implantation, fetal growth, and lactation in animals^21, 59–61^. Also, low birth weight, reduced litter sizes, and lower survival rates are adverse pregnancy outcomes induced by gestational stress^62–66^. In the present study, average weight gain was lower in the stressed groups and in particular, it was significantly lower in the AS group compared to the other groups. This lower weight gain could be the result of both abortion (resorbed embryos) and implantation rate in the AS group. In addition to significantly higher resorption rate in the AS group, the average number of implantations was lower in the AS group than the PS and the control groups, although the difference was not significant. In this regard, Zamora *et al*.^67^ reported significantly decreased blastocyst production in mice exposed to increased housing rack noise. The mean noise was 80.4 dBC in the ventilated rack and 69.2 dBC in the static rack, and the authors showed a reduced blastocyst count in bred C57BL/6 donor mice housed in ventilated cages.

Furthermore, the number of the live pups observed on the first PD in the current study was significantly lower in the AS group than the other groups, and it was slightly decreased on the second PD. It was not possible in this study to determine the pups’s health status at that age point. The number of live pups, however, did not change in the next post-delivery days. In addition, investigating uterus receptivity by counting the number of resorbed signs and implantation sites^14, 16^ indicated a remarkably higher number of resorbed signs in the AS group than the control group. The number of survived pups in the AS group was also significantly lower than the number of implantation sites compared with the other groups. However, no significant difference was observed among the three groups in terms of the number of implantations. In accordance with the behavioural findings, the reduced uterus receptivity and the low rate of survived pups in the AS group indicated a stronger effect of the AS than the PS exposure in the dams. An altered immune response due to gestational stress is the most likely reason for the high rate of spontaneous abortion in mammals^14, 16, 22^. Subjecting pregnant mice to a brief period of ultrasonic- acoustic stress during gestation increases the abortion rate owing to increased levels of TNFα and transforming growth factor β2 (TGFβ2). Both factors decrease the immune system activity and negatively affect the uterus receptivity^14, 16, 20^.

While the adverse effect of stress on reproduction by influencing the implantation of embryos and increasing the rate of abortion has been previously addressed^16, 20, 21^, there is no report of eating or killing pups as a result of gestational stress. Our results indicated that AS reduced the number of pups on the first early PD. It is difficult to determine how much of this loss is specifically the result of killing pups, because the health condition of eaten pups was unknown. Although it was not investigated in the current study, changes in nest building can provide a general view of a mouse’s anxiety state, and is an indication of psychological distress^68^ in gestationally stressed dams. Thus, assessing nest structure and quality in a systematic manner^69, 70^ in future studies would be a useful way to test anxiety-like behaviour in stressed mice during peripartum and the early postpartum days.

Gestational stress can produce long-lasting dysfunction of the HPA and hypothalamo–gonadal (HPG) axes^71, 72^, and can elevate corticosterone levels in dams and fetuses^73^. Brummelte and Galea^74^ demonstrated that injecting corticosterone into pregnant dams negatively affected postpartum maternal care and decreased the time spent in nursing pups. The 11þ-hydroxysteroid dehydrogenase-2 (11þ-HSD-2) is an enzyme that is present in the placenta and central nervous system, and converts extra levels of corticosterone to relatively inactive products in normal pregnancies. In mid-gestation the expression of this enzyme is greatly decreased in the rodents^75–77^, and it seems that stress during pregnancy can decrease it further. While the loss of pups in our study could be associated with maternal stress and excess levels of corticosterone in the dams, female testosterone is another possible mechanism for this behaviour. Infanticide by adult males is modulated by the presence or absence of testosterone, and castration can reduce this behaviour^78^. Furthermore, depending upon the dose and duration of testosterone treatment, 35% to 100% of the rodent dams can be induced to kill their pups^79^. Therefore, investigating how hormones and other biochemical, epigenetic, or neurogenic factors change in the female mice under gestational AS needs to be addressed in future studies.

The PS paradigm used in our study failed to induce anxiety-like behaviour. Previous studies used a period of restraint ranging from 30 or 45 min three times a day for a week, either as a single stressor or combined with other stressors^80–86^. A recent study used a 30 min restraint stress paradigm for three times a day between GDs 5 and 19 in mice, affected the dams’ depressive-like, but not their anxiety-like behaviour^86^. Due to procedural and organizational demands, we were unable to apply a longer period of restraint stress in the present study. Hence, our results could be affected by the shorter period of stress exposure than previous studies. Thus, it seems that replication of our methodology in the same mouse strain with longer PS exposure could provide further information on the effects of stress on pregnant mice. Contrary to the effects of PS, AS induced greater anxiety-like behaviour along with significant alterations in pregnancy outcome.

Loud noises are usually a source of information, such as warnings for risks, strains, or dangers in many species, and they readily induce HPA, autonomic, and behavioural responses^87^. Also, long term exposure to high levels of corticosterone downregulates the glucocorticoid receptors and makes a vicious endocrine cycle of increased corticosterone levels and enhanced responsiveness to stress. On the other hand, the glucocorticoid receptors of the organ of Corti that respond to both systemic stress and acoustic stimulation play an important role in the mechanism of down regulation of glucocorticoid receptors^88^. Sleep deprivation and disruption is also a factor that might influence our findings in the AS group, since it can activate the stress-responsive regulatory systems^89^ and significantly affect corticosterone and adrenocorticotropic hormone secretion^90^. In addition, noise exposure leads to increased aggression^91^, interpersonal animosity^92, 93^, and curtailment of prosocial behaviour in humans^94, 95^. These can be taken as indications of hostility and generalized aggression^96^. These findings from human studies along with our findings here, highlight the importance of further studies on noxious effects of gestational AS on brain and behaviour in human and laboratory animals in the future.

There are some procedural limitations that should be considered in this study. First, although we used well-known protocols for exposing animals to AS and PS, these types of experimental stressors are different to some extent than those typical auditory and physical stress observed in real life. Thus, experimental conditions that provide more analogous models of stress to natural stressors are recommended for future studies. The second limitation of our finding is that we did not measure corticosterone levels, which might have allowed us to compare the relative severity of the two different types of stressors. Nevertheless, even in the absence of hormonal indicator of the stress response, we believe that the alterations found in the present study demonstrates a difference in the effects of the two stressors. In addition, investigating the health condition of the AS pups who did not survive after birth, as well as exploring maternal behaviour of the dams during the late peripartum and the early postpartum periods^68, 70^ can help to make clear the influence of pups health as well as maternal anxiety on loss of pups in the early PD. Finally, pre-exposure to an elevated platform as a stressor for the PS group could influence the later behaviour of the PS dams in both EPM and BBT. It might cause an attenuating or boosting effect on the results that can be examined in the future by adding another PS group free of elevated platform stress.

## Methods

### Animals

All experiments were carried out in accordance with the Canadian Council of Animal Care and approved by the University of Lethbridge Animal Care Committee. All animals were given access to food and water ad libitum and were maintained on a 12:12-h light:dark cycle in a temperature-controlled breeding room (21 °C) with less than 66 ± 2 dBC room noise level. Thirty female C57BL/6 mice were individually mated with thirty male C57BL/6 mice in standard shoe-box cages. Mice were singly housed once the pregnancy was confirmed. The rate of weight gain during pregnancy (grams), duration of pregnancy (days), number of the live pups on the first PD, and number of the survived pups during the early postnatal period (P0-P4) were calculated for each mouse.

#### Recordings of gestational length

Female mice between 8 to 11 weeks of age were housed with a male at 4:00 pm. The female mice were assessed three hours later at 7:00 pm and the next morning for breeding signs such as sperm plug and red/swollen vaginal opening^28^. The female mice were considered possibly pregnant only if the breeding signs were present on both observations. Female mice with a negative sign of the breeding were not paired with male mice for the period of 11 days until the lack of pregnancy was confirmed. The weight gain of the female mice was followed every day to confirmed pregnancy. On the gestational day (GD) 11, a weight gain of at least 3.5 g usually signifies conception has occurred. This method allows a determination of the length of gestation with a 0.5-day precision.

### Experimental design

Pregnant mice were randomly assigned into either auditory or physical stress groups or a control group. We administered gestational stress during days 12–16 of gestation because the significant section of the corticogenesis process occurs between embryonic days 12–16^2, 97^.

#### Gestational AS group

On gestational days (GDs) 12, 14, and 16 a pregnant mouse was transferred into a standard cage and moved to a sound chamber. Dams (n = 10) were presented with an intermittent 1 sec tone (3 KHz; 90 dB)^14, 16, 19^ with an inter-stimulus interval of 15 seconds for 24 hrs period starting at 8:00 am.

#### Gestational PS group

Two stressors, restraint and elevated platform (EP), were applied daily from GDs 12 through 16. For restraint, mice (n = 10) were maintained in a transparent Plexiglas container (5 cm inner diameter), 20 minutes per day at 10:00 am. The container maintained the mice in a standing position without compression of the body^98, 99^. For the EP stressor, each mouse was placed on an elevated platform (1 m height, 21 × 21 cm), 30 minutes twice a day at 9:00 am and 3:00 pm^4, 38^.

#### Control group

There were two sets of control animals: one served as a control for AS dams and another was a control for PS dams. In AS control group, pregnant mice (n = 5) on GDs 12, 14, and 16 were individually transferred into a standard cage and moved to the sound chamber. The dams were left undisturbed for 24hrs starting at 8:00am. In PS control group, pregnant mice during GDs 12–16 (n = 5) were transferred daily from their home cage to the same testing room used for the PS group. The dams left undisturbed for 20 or 30 minutes (depending on the type of stressor) and later were returned to their home cages.

### Behavioural assessments

We used three behavioural tests after weaning (21–23 days after birth) to measure the effect of gestational stress on the dams’ cognitive and balance performance. The behavioural tests began one day after weaning and were completed 4 days later. NOR, EPM, and BBT tests were conducted respectively on separate days every morning between 9–10 am.

#### Novel object recognition (NOR) test

Each mouse was placed in an open-field arena (47 cm width × 50 cm length × 30 cm height) made of white Plexiglas. In the first trial, the mouse was placed in the arena with two identical objects and explored the field for 5 min. The animal was removed and placed in a transport box for 3 min, and one of the objects was randomly replaced with a new object. The mouse was then returned to the arena, and the animal’s exploration was filmed (30 frames/second) for 3 minutes. Time spent with each object was only calculated during the second session. If the nose of the mouse was within 1 cm of the object, it was considered to be in contact with an object^38, 39^. The ratio of time spent with the old compared to the new object was calculated by subtracting times spent with ‘old’ from the new object divided by the total time spent for exploration^100^.

#### Elevated plus maze (EPM) test

The EPM apparatus consisted of 3 main components: a base (48 cm), two open arms (5 cm × 27 cm), and two closed arms (5 cm × 27 cm × 21 cm). The apparatus, made of black Plexiglas®, was housed in a well-lit empty testing room. The camera for filming was placed at the end of an open arm slightly above the maze. Mice were placed with their front paws in the centre area and facing a closed arm. Each mouse was filmed (30 frames/sec) for 5 min and scored for time spent in open arms and number of entries to open arms^32, 42^. Animals were considered in an arm when the front half of the body was within the arm^34, 101^. The center zone connecting all arms was excluded^33^.

#### Balance beam test (BBT)

Mice were required to traverse an elevated, narrow aluminium beam (1 cm diameter, 100 cm long and 50 cm above a foam pad to cushion falling mice) to reach an enclosed escape box. Mice were first trained (4–5 trials) and were tested (3 trials) on the next day. We calculated the mean latency (sec), distance travelled, number of foot slips, number of turns, and number of falls across the 3 testing trials^102^. Only the BBT needed two test days, one day for the training trials and the following day for the testing trials.

#### Mouse uterus receptivity

Upon completing the behavioural tests, all dams were euthanized, 28–40 days after parturition and their uteri were removed. Uterus receptivity was investigated by counting the number of implantation sites and reabsorption signs^17^. The placenta is a flattened circular organ in the uterus of pregnant mammals, nourishing and maintaining the fetus through the umbilical cord^103^. In Figure 4F, each dark spot marked by a black line reflects an implantation site or the place of an embryo connected to the placenta; and each pale sign marked by a red line, indicates a resorbed sign or the place of an embryo aborted or disconnected from the placenta^17^. To measure the effect of gestational stress on the number of surviving pups, we periodically observed each pregnant mouse inside her standard shoe-box cage and looked for the new pups or any changes in the number of pups within 3 days before the estimated birth time, in order to determine if there were any preterm deliveries, as well as 5 days after the parturition. The number of lost pups during the early postnatal period (P0–P4) was calculated by subtracting the number of surviving pups from the number of identified implantation sites in the uteri.

#### Statistical analysis

All statistical analyses were done using SPSS Statistics 24.0 using an alpha level of 0.05. We used the Kolmogorov–Smirnov test for normally distributed data. To test for differences between the three studied groups for age, different parameters of the three behavioural tests, as well as the pregnancy outcome and the uterus receptivity including weight gain due to pregnancy (gram), pregnancy duration (day), number of the live pups on the first PD, number of surviving pups, number of the resorbed signs, and number of the implantation sites we used Multivariate analysis of variance (MANOVA). To compare the number of live pups on the first and the second PDs as well as the number of surviving pups and the number of implantation sites a repeated measures ANOVA was used. For multiple comparisons of group means in each measurement, the Tukey post-hoc test was performed.
